# Analyzing the Influence of Imaging Resolution on Polarization Properties of Scattering Media Obtained From Mueller Matrix

**DOI:** 10.3389/fchem.2022.936255

**Published:** 2022-07-12

**Authors:** Conghui Shao, Binguo Chen, Honghui He, Chao He, Yuanxing Shen, Haoyu Zhai, Hui Ma

**Affiliations:** ^1^ Department of Physics, Tsinghua University, Beijing, China; ^2^ Guangdong Research Center of Polarization Imaging and Measurement Engineering Technology, Shenzhen Key Laboratory for Minimal Invasive Medical Technologies, Institute of Optical Imaging and Sensing, Shenzhen International Graduate School, Tsinghua University, Shenzhen, China; ^3^ Institute of Biopharmaceutical and Health Engineering, Tsinghua Shenzhen International Graduate School, Tsinghua University, Shenzhen, China; ^4^ Department of Biomedical Engineering, Tsinghua University, Beijing, China; ^5^ Department of Engineering Science, University of Oxford, Oxford, United Kingdom

**Keywords:** Mueller matrix microscope, Monte Carlo simulation, fibrous structure, imaging resolution, polarization

## Abstract

The Mueller matrix contains abundant micro- and even nanostructural information of media. Especially, it can be used as a powerful tool to characterize anisotropic structures quantitatively, such as the particle size, density, and orientation information of fibers in the sample. Compared with unpolarized microscopic imaging techniques, Mueller matrix microscopy can also obtain some essential structural information about the sample from the derived parameters images at low resolution. Here, to analyze the comprehensive effects of imaging resolution on polarization properties obtained from the Mueller matrix, we, first, measure the microscopic Mueller matrices of unstained rat dorsal skin tissue slices rich in collagen fibers using a series of magnifications or numerical aperture (NA) values of objectives. Then, the first-order moments and image texture parameters are quantified and analyzed in conjunction with the polarization parameter images. The results show that the Mueller matrix polar decomposition parameters diattenuation *D*, linear retardance *δ*, and depolarization *Δ* images obtained using low NA objective retain most of the structural information of the sample and can provide fast imaging speed. In addition, the scattering phase function analysis and Monte Carlo simulation based on the cylindrical scatterers reveal that the diattenuation parameter *D* images with different imaging resolutions are expected to be used to distinguish among the fibrous scatterers in the medium with different particle sizes. This study provides a criterion to decide which structural information can be accurately and rapidly obtained using a transmission Mueller matrix microscope with low NA objectives to assist pathological diagnosis and other applications.

## Introduction

The polarization imaging approach has shown broad application potential in biomedical studies in recent years for its advantages of being noninvasive, label free, and sensitive to subwavelength structures (Alali and Vitkin, 2015; Qi and Elson, 2017; He C et al., 2019; He et al., 2021). The Mueller matrix, which characterizes the change of polarization state of light after light–matter interaction, contains rich microstructural information about the medium (Chen et al., 2020; Hu et al., 2020). However, it is often difficult to obtain specific microstructural information through individual Mueller matrix elements (He et al., 2022; Li et al., 2022). To further disentangle the information encoded in the Mueller matrix, the Mueller matrix polar decomposition (MMPD) method (Lu and Chipman, 1996; Ghosh et al., 2008) was proposed and prevalently used in the biomedical studies (Morio and Goudail, 2004) to derive a group of polarization parameters with clear physical meanings. These parameters can be applied on characterizing structure features of various abnormal tissue samples, such as liver fibrosis (Wang et al., 2016; Meng et al., 2021; Yao et al., 2022), breast ductal carcinoma (Dong and Qi, 2017; He H et al., 2019; Dong et al., 2021a), skin cancer (Steven et al., 2002; Jacques et al., 2002; Wood et al., 2009; Du et al., 2014), colon cancer and inflammatory bowel disease, cervical cancer (Sun et al., 2014; Dong et al., 2021b), and oral cancer (Chung et al., 2007). In addition, polarization imaging methods are particularly sensitive to fibrous structures in tissues, such as the location, density, and orientation arrangement of fibers at different stages of pathological tissue development (Dong and Qi, 2017; He H et al., 2019). Thus, the Mueller matrix-derived parameters can be used for differential diagnosis of Crohn’s disease and intestinal luminal tuberculosis by various features in the distribution of fibers around the granuloma (Liu et al., 2019). Moreover, Mueller matrix polarimetry has also shown potential to quantitatively distinguish among different types of fibrous tissues, such as collagen fibers, connective tissues, and muscle fibers (Zhai et al., 2022).

For optical methods, higher resolution images provided by a high numerical aperture (NA) objective often give us more detailed microstructural information about the sample. However, the field of view (FOV) of the image provided by a high NA objective is smaller compared to that provided by a low NA objective, which means that the acquisition speed is slow for high-resolution images. When applied to clinical detection, the imaging speed should be considered in addition to the impact of imaging resolution. Some recent researches have shown that the polarization imaging method can better preserve the microstructural information of the sample when imaging resolution decreased compared to unpolarized optical imaging methods (Shen et al., 2020; Liu et al., 2021; Chen et al., 2022; Yao et al., 2022). It indicates that adopting the polarization imaging method can well balance the requirements of imaging resolution and FOV, to acquire the micro- and even nanostructural (Dong et al., 2020; Chen et al., 2021a; Wang et al., 2021; Fang et al., 2022) properties of the scattering medium quickly using a relatively low NA objective. In addition, to further obtain more quantitative information, many studies combined polarization parameters and image texture methods together to better distinguish characteristic tissue structures (Liu et al., 2019; Zhai et al., 2022). However, recent studies mainly investigated the influence of imaging resolution on linear retardance parameters reflecting orientation and density information of the fibrous structures, with less analysis of the influence on other Mueller matrix derived parameters revealing such as particle size information that may exist at nanoscale. Here, to further analyze the comprehensive effects of imaging resolution on polarization properties obtained from the Mueller matrix, namely diattenuation, linear retardance, and depolarization, we, first, measure the Mueller matrices of unstained rat dorsal skin tissue slices rich in collagen fiber using a transmission Mueller matrix microscope. The MMPD parameters *D*, *δ*, and *Δ* images of the sections are calculated at a series of magnifications of objectives with 4×/NA 0.10, 10×/NA 0.25, 20×/NA 0.40, 40×/NA 0.65, and 60×/NA 0.80. Then, the first-order moments and image texture parameters are quantified and analyzed in conjunction with the polarization parameter images. The results show that the MMPD parameters *D*, *δ*, and *Δ* images obtained using a low NA objective, such as 10×/NA 0.25, retain most of the structural information of the sample, and can provide fast imaging speed. In addition, the analysis based on the scattering phase function calculation of cylinders and the Monte Carlo simulation based on the cylindrical scatterers reveal that diattenuation parameter *D* images with different imaging resolutions are expected to be used to distinguish among the fibrous scatterers in a medium with different particle sizes. This study provides a criterion to decide which structural information can be accurately and rapidly obtained using a transmission Mueller matrix microscope with low NA objectives to assist pathological diagnosis and other applications.

## Materials and Methods

### Setup and Samples

**FIGURE 1:**
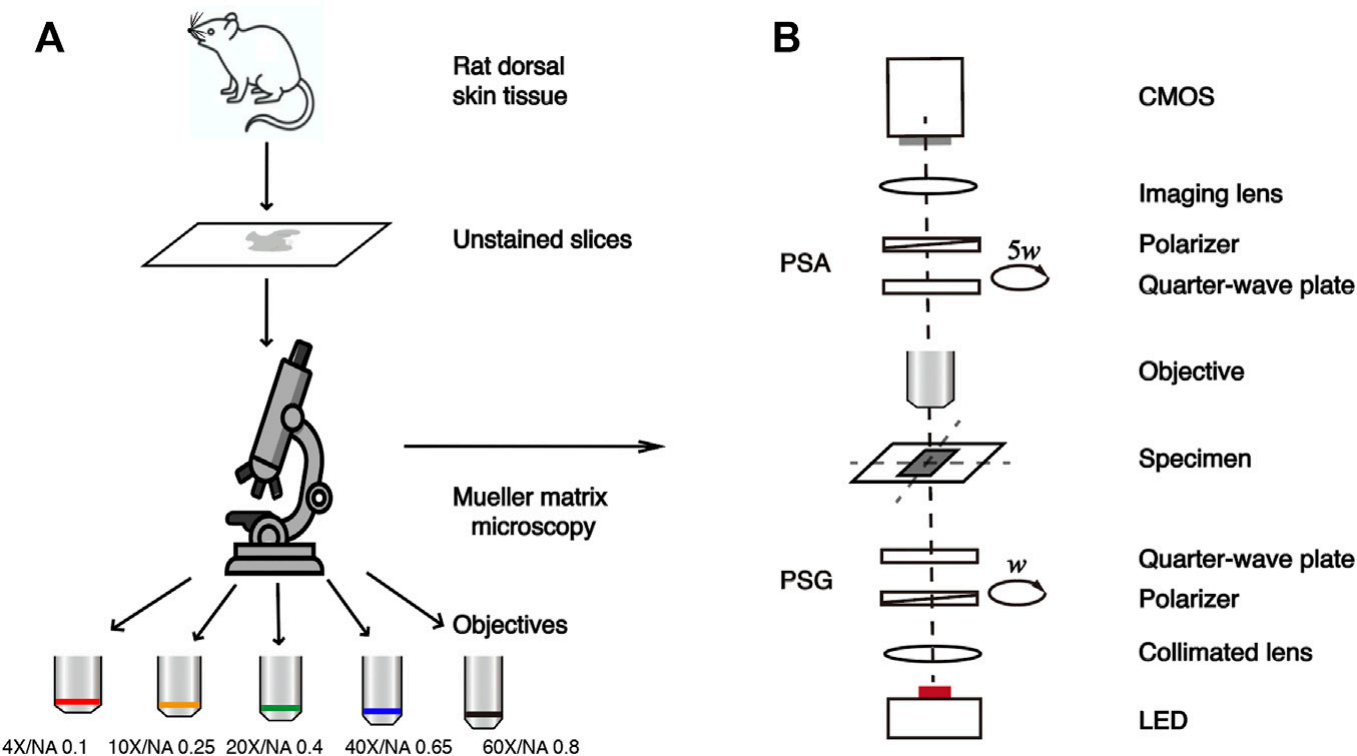
Schematic of the study. **(A)** Flow chart of the experiment. **(B)** Schematic of the transmission Mueller matrix microscope.

Figure 1 shows the schematic of this study. The transmission Mueller matrix microscope used in this study is based on the dual-rotating quarter-wave plate method (Goldstein, 1992). As shown in Figure 1B, the illuminating light from the light-emitting diode (Cree, 3W, 633 nm, Δλ = 20 nm) is collimated by a lens, and then passes through the polarization states generator (PSG). Light carrying different polarization states transmits the sample and is collected by the objective lens at different magnifications. It is then analyzed by the polarization states analyzer (PSA) and focused on the CMOS camera (MV-CA016-10UM, 1,440 × 1,080 pixels, 12-bit, 3.45 μm × 3.45 μm pixel size, Hikvision, China) through an imaging lens. PSG and PSA have similar structures, both consisting of a linear polarizer fixed in the horizontal direction (extinction ratio 1000:1, Daheng Optics, China) and a rotatable quarter-wave plate (Daheng Optics, China) as shown in Figure 1B. In this setup, both PSG and PSA are driven to rotate thirty times by the servo motor drivers (PRM1Z8E, Thorlabs, United States) with the fixed rates ω and 5ω, respectively. Then the Mueller matrix elements can be calculated by using the Fourier coefficients (Azzam, 1978; Chenault et al., 1992). The Fourier series intensities are given as

$$I=\alpha_{0}+\sum_{n=1}^{12}\left(\alpha_{n}\cos\;n\omega t+\beta_{n}\cos\;n\omega t\right),$$ (1)

where *α*_*n*_ and *β*_*n*_ are the Fourier coefficients. Before the measurement, the Mueller matrix microscope was calibrated using some standard samples such as air, polarizers, and retarders in different directions. The error is within 1% and the detailed calibration procedure can be found in (Zhou et al., 2018).

In this study, the samples are 6-μm-thick dewaxed, unstained slices of rat dorsal skin tissues, provided by the Experimental Research Center, China Academy of Chinese Medical Sciences. The rat dorsal skin tissue contains skeletal muscle fibers, connective tissues, and collagen fibers (Sun et al., 2018; Chen et al., 2021b; Zhai et al., 2022). Here we selected a total of 27 tissue regions rich in collagen fibers, which have prominent optical anisotropy as the experimental samples for the Mueller matrix measurement. The flow chart of this study is illustrated in Figure 1A.

### Mueller Matrix Polar Decomposition

The polarization state of light can be changed after the scattering process, and the micro- and nanostructural information of the scattering sample can be reflected by measuring the Mueller matrix, which is a comprehensive description of polarization-related optical properties of the medium. However, the physical meanings of individual Mueller matrix elements are not clear. To develop associations between structural features and the elements, the MMPD method decomposes a Mueller matrix into three submatrices of major polarization properties: diattenuation matrix *M*_*D*_, retardance matrix *M*_*R*_, and depolarization matrix *M*_*Δ*_ as shown in Eq. 2. It is noted that *M*_ij_ (i,j = 1,2,3,4) in Eq. 3 represents the corresponding Mueller matrix elements before decomposition, while *M*_*R*_(i,j) (i,j = 2,3) and *M*_*Δ*_ in Eqs (4) and (5) represent the matrix elements in the 4 × 4 retardance matrix and the depolarization matrix, respectively. Here, the retardance can be further decomposed into circular retardance and linear retardance (Ghosh, Wood, and Vitkin, 2008), where *δ* is the magnitude of linear retardance. In this study, we adopt the MMPD-derived parameters *D*, *δ,* and *Δ*, which reflect diattenuation, the value of linear retardance, and the depolarization of tissue samples, respectively.

$$M=M_{\Delta }M_{R}M_{D},$$ (2)

$$D=\sqrt{M_{12}^{2}+M_{13}^{2}+M_{14}^{2}},$$ (3)

$$\delta =\mathrm{co}s^{-1}\left\{{\left[{\left(M_{R}\left(2,2\right)+M_{R}\left(3,2\right)\right)}^{2}+{\left(M_{R}\left(3,2\right)+M_{R}\left(2,3\right)\right)}^{2}\right]}^{\frac{1}{2}}-1\right\},$$ (4)

$$\Delta =1-\frac{\left|\mathrm{tr}\left(M_{\Delta }\right)-1\right|}{3}.$$ (5)

### Image Analysis Method

During the Mueller matrix measurement of tissue sections, to ensure that the same area is detected by different magnification objectives, we only rotate the objective without changing the position of tissue slices. The field of view difference is calibrated by measuring the coordinate positions of the standard sample (calibration plate) in the same area under different magnification objectives.

**FIGURE 2:**
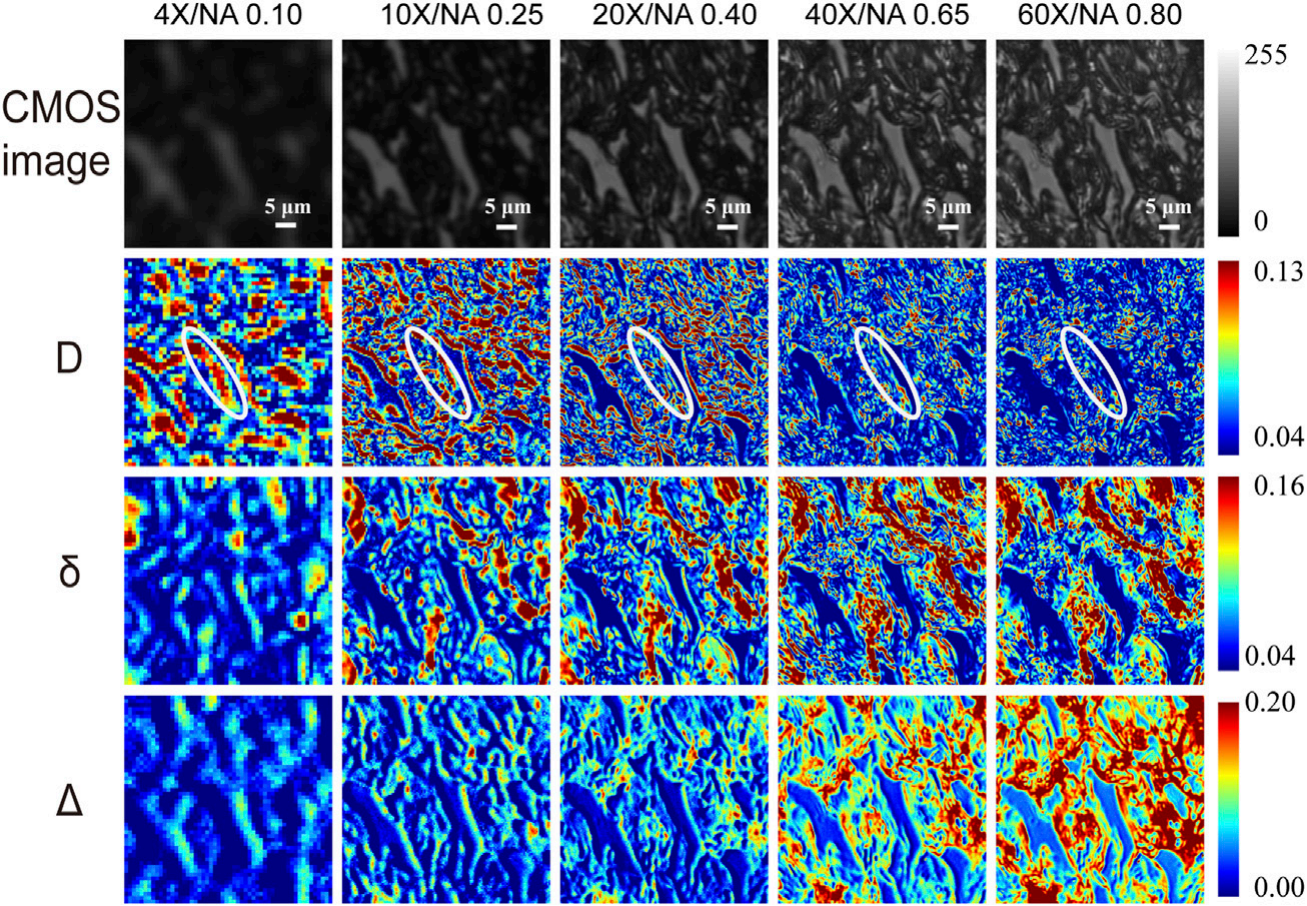
The first row shows the unpolarized light intensity images of the unstained tissue slice under different magnification objectives (4×/NA 0.10, 10×/NA 0.25, 20×/NA 0.40, 40×/NA 0.65, and 60×/NA 0.80). The second to fourth rows show MMPD parameters *D*, *δ*, and *Δ* images of the same region under different magnification objectives. Where the area marked by the white elliptical line in the D images is an example of a coarse fibrous structure that is not clear under high NA but obvious under low NA. The unit for *δ* is radian angle. The white scale bar is 5 μm.

To quantitatively investigate the effect of different magnifications on polarization microscopic imaging, we first compare MMPD *D*, *δ*, and *Δ* parameters images at the magnification objectives of 4×/NA 0.10, 10×/NA 0.25, 20×/NA 0.40, 40×/NA 0.65, and 60×/NA 0.80, as shown in Figure 2. Then, to further quantify the changes of the MMPD parameters at different magnification objectives, we use two first-order statistical moment parameters, the Mean and Entropy shown in Eqs (6) and (7) for evaluations, where *p*(*z*_*i*_) represents the ratio of the number of pixels with the value of *z*_*i*_ to the total number of the pixels.

$$\mathrm{Mean}=\underset{i}{\sum }z_{i}p\left(z_{i}\right),$$ (6)

$$\mathrm{Entropy}=-\underset{i}{\sum }p\left(z_{i}\right)\mathrm{lo}g_{2}p\left(z_{i}\right).$$ (7)

Additionally, we also choose the gray scale co-occurrence matrix (GLCM) method (Haralick et al., 1973), which has been demonstrated as a powerful tool for the Mueller matrix imaging results analysis in recent studies (Shen et al., 2020; Liu et al., 2021; Zhai et al., 2022), for the MMPD parameters comparisons. Here, the GLCM parameters Contrast, Correlation, Energy, and Homogeneity are adopted as shown in Eqs 8–12. For the analysis, the ranges of gray value for the parameters *D*, *δ,* and *Δ* images are normalized to [0,255], the gray levels *N*_*g*_ was set as 64, the inter-pixel displacement *d* was set to 1, 3, 5, 11, and 15 under 4×/NA 0.10, 10×/NA 0.25, 20×/NA 0.40, 40×/NA 0.65, and 60×/NA 0.80 objectives, respectively, to compensate the differences induced by the different FOV. *p(i, j)* is the relative frequency of two adjacent pixels (gray level *i* and *j*, respectively) separated by an inter-pixel displacement (*d*) occurring in a specific direction on the image. *μ*_*x*_, *μ*_*y*_, σ_x_, and σ_y_ are the mean and standard deviations of *p*_*x*_ and *p*_*y*_, respectively. Contrast characterizes the local variation in GLCM, the higher the Contrast value, the better the ability to distinguish the various components of the image. Correlation is a measure of the correlation of a pixel with its neighboring pixels in the whole image. Energy characterizes the joint probability of the occurrence of a given pixel pair, reflecting the order in the image, a higher Energy value means a more uniform texture of the image. Homogeneity returns a value that measures the closeness of the distribution of elements in the co-occurrence matrix, reflecting the order of the local image.

$$p_{x}\left(i\right)=\overset{N_{g}}{\underset{j=1}{\sum }}p\left(i,j\right)\;p_{y}\left(j\right)=\overset{N_{g}}{\underset{j=1}{\sum }}p\left(i,j\right),$$ (8)

$$\mathrm{Contrast}=\overset{N_{g}-1}{\underset{n=0}{\sum }}n^{2}\left\{\overset{N_{g}}{\underset{i=1}{\sum }}\overset{N_{g}}{\underset{j=1}{\sum }}p\left(i,j\right)\text{|}\left|i-j\right|=n\right\},$$ (9)

$$\mathrm{Correlation}=\frac{\sum_{i}\sum_{j}\left(\mathrm{ij}\right)p\left(i,j\right)-\mu_{x}\mu_{y}}{\sigma_{x}\sigma_{y}},$$ (10)

$$\mathrm{Energy}=\underset{i}{\sum }\underset{j}{\sum }p{\left(i,j\right)}^{2},$$ (11)

$$\mathrm{Homogeneity}=\underset{i}{\sum }\underset{j}{\sum }\frac{1}{1+{\left(i-j\right)}^{2}}p\left(i,j\right).$$ (12)

### Monte Carlo (MC) Simulation

To better understand the relationship between the micro- and nanostructural features observed at different magnifications and the Mueller matrix derived parameters, we use the MC simulation program based on the cylinder scattering model (CSM) developed in our previous studies (Yun et al., 2009; Guo et al., 2013; Li et al., 2016) to track the trajectories and polarization states of scattered photons as they propagate in the skin tissues (Chen et al., 2017; Dong and He, 2017). The detailed parameters used in MC simulations will be introduced in the following sections.

## Results

To further analyze the influence of different magnifications, or the imaging resolutions on the polarization information acquired by Mueller matrix derived parameters, in this section the first-order statistical moments Mean and Entropy as well as the GLCM parameters Contrast, Correlation, Energy, and Homogeneous are used to quantify the MMPD *D*, *δ*, and *Δ* parameters.

Also, to compare the structural evaluation ability under different magnifications, the correlation of the Mean values between each of the two objectives is analyzed to characterize the similarity of the structure contained in the MMPD parameters. A higher R-value (the Pearson correlation coefficient) indicates a stronger correlation between the two sets of data, or in other words the more similar information contained in Mueller matrix images obtained by the two objectives. In addition, for a deeper understanding of the experimental results, we use the MC simulation, in which the fibers are simplified to infinitely long cylindrical scatters with different particle sizes and phase functions, to analyze the influence of collecting angle on Mueller matrix derived parameters.

Figure 2 shows the microscopic imaging results of a normal rat dorsal skin region that is abundant in collagen fibers at different magnifications. The first row from left to right shows the unpolarized light intensity images of unstained tissue sections under 4×/NA 0.10, 10×/NA 0.25, 20×/NA 0.40, 40×/NA 0.65, and 60×/NA 0.80 objective cases, respectively. The second, third, and fourth rows show the *D*, *δ,* and *Δ* images of 4×, 10×, 20×, 40×, and 60× for the same region, respectively. We can observe from the first row of Figure 2 that unpolarized images obtained at high magnifications are significantly sharper and richer in microstructural information than those obtained at low magnifications. It is confirmed that a better imaging resolution can provide more structural details when using unpolarized light microscopy. However, for Mueller matrix microscopic imaging, our previous studies indicated that some structural information contained in the linear retardance *δ* parameter image is preserved well with the decline of imaging resolution or objective magnifications (Shen et al., 2020). We can also see from Figure 2 that when the magnification decreases from 60× to 4×, the changes in MMPD parameters are different. For instance, for *δ* and *Δ* images, with the decreasing resolution, tiny fibrous structures become less obvious and lack some detailed information. On contrary, the regions containing coarse fibrous structures can be observed at low magnification, and we can see that they are composed of tiny fiber bundles at high magnification. However, this does not prevent us from being able to roughly obtain structural information such as fiber density and location from the low-resolution polarization images. It can also be noticed that the fibrous structures are not obvious in high magnification parameter *D* images, and detailed information on the fibers can be observed in low-resolution *D* images. For *D* images, the coarse fibrous structures marked with white elliptical lines in Figure 2, as an example, have lower values at high magnification, which are not obvious at high NA. The coarser fibrous structures are more visible at low magnification, and only some tiny fibrous structures can be observed at high NA. The results shown in Figure 2 indicate that when tissue samples with certain polarization properties are analyzed, a criterion for choosing an appropriate objective magnification or NA value to achieve a balance between FOV and enough feature information extraction is crucial.

### Analysis of Diattenuation

**FIGURE 3:**
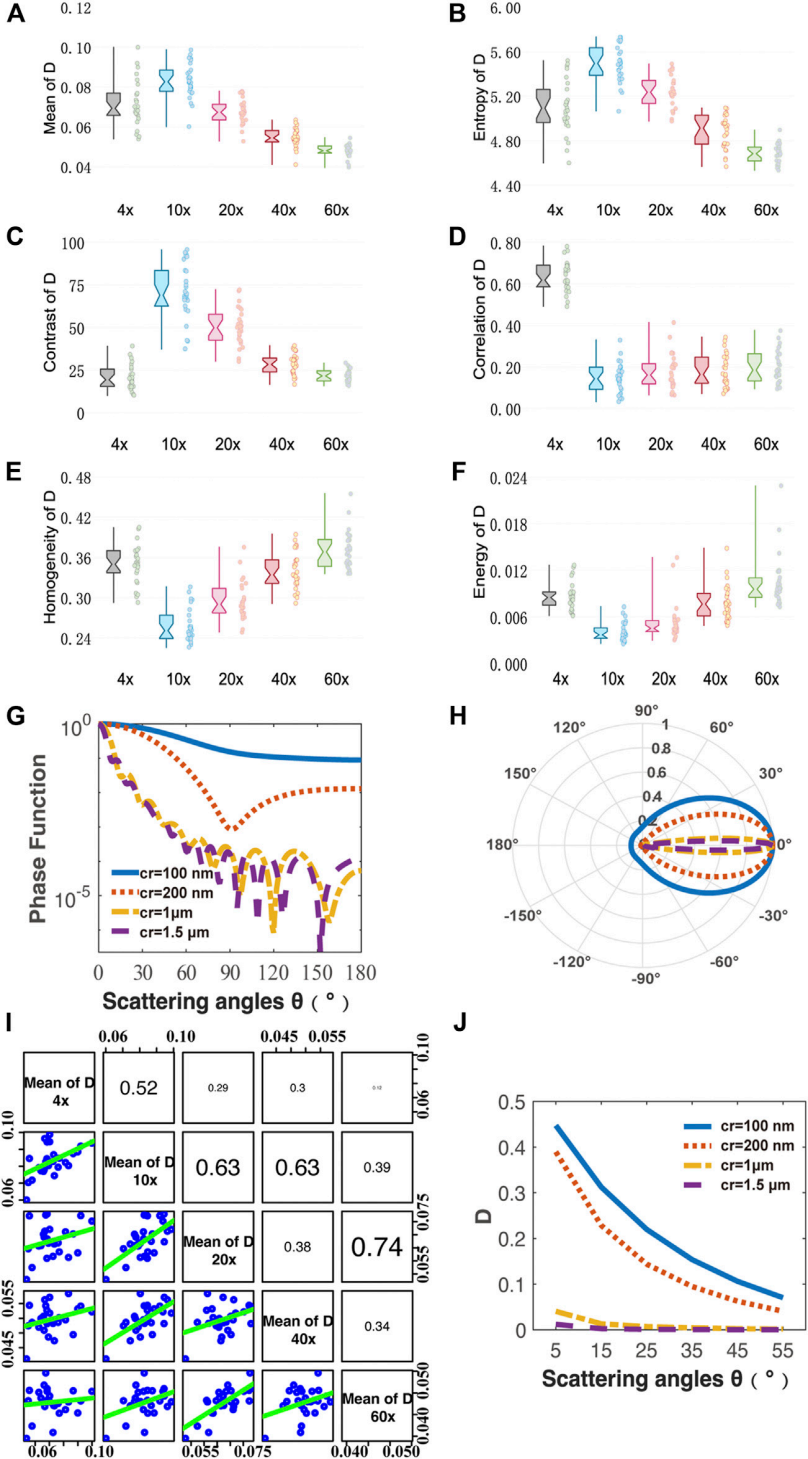
Quantitative analysis of MMPD parameter *D* images under different magnification objectives (4×/NA 0.10, 10×/NA 0.25, 20×/NA 0.40, 40×/NA 0.65, 60×/NA 0.80). **(A–F)** Box plots of Mean, Entropy, Contrast, Correlation, Homogeneity and Energy. **(G,H)** Phase functions for cylinder scatterers with radius of 100, 200, 1, and 1.5 μm. **(I)** The scatter-plot matrix illustrates the general correlation among the Mean value of D under different magnification objectives (4×/NA 0.10, 10×/NA 0.25, 20×/NA 0.40, 40×/NA 0.65, 60×/NA 0.80). The variables are written in a diagonal line from top left to bottom right. On the right of the diagonal are the correlation coefficient *R* between two pairs of mean of *D* at different magnifications, with the larger the font size, the higher the correlation. On the left side of the diagonal is the scatter-plot matrix, with smooth green trend line to illustrate the underlying relationship. **(J)** Monte Carlo simulation results of the parameter *D* using the cylinder scatterer model of different radius: 100, 200, 1, and 1.5 μm. The horizontal axis represents different scattering angle from 5° to 55°.

First, we analyze the MMPD diattenuation parameter *D* as shown in the second row of Figure 2. In previous studies, it was shown that *D* can also be used for describing the changes in the fibrous microstructures in tissues (Swami et al., 2006; Fan and Yao, 2013; Menzel et al., 2019). Meanwhile, it is shown in Figure 2 that the changing trend of *D* is different from parameters *δ* and *Δ*. Hence, in this section, we first analyze the variation of parameter *D* with the objective magnification. As we can see in Figure 3, both the Mean (Figure 3A) and Entropy (Figure 3B) values of *D* initially increase and then gradually decrease as the objective magnification becomes larger, reaching their peak values at 10×. For GLCM parameters, the variation trend of Contrast (Figure 3C) is similar to that of Mean and Entropy. It means that the fibrous structure information contained in the *D* image first increases and then gradually decreases. However, the values of the remaining three parameters: Correlation (Figure 3D), Homogeneous (Figure 3E), and Energy (Figure 3F) decreased significantly from 4× to 10×, and then increase slowly with the increasing objective magnification. It indicates that from 10× to 60× the image texture of the obtained *D* parameter becomes more uniform, similar, and ordered. The possible reason is that there may be the loss of image details for large magnifications, which can result in smooth and blurry image textures. It can also be demonstrated in Figure 2 that in parameter *D* images some fibers with small particle sizes are gradually unobservable with the increasing magnification, especially when the 60× objective was used. It may be induced by a significant decrease in *D* of the tiny cylinder at high magnification, where the fiber structures are no longer apparently leading to a decrease in imaging contrast at high magnification and the image texture becomes smooth and blurred. To testify, we obtain the phase functions of the infinitely long cylindrical scatterers (Kienle et al., 2003; Yun et al., 2009) with different particle sizes as shown in Figures 3G,H, in which the zenith angle is 90°. Here, the cylinders with a radius of 100 nm (blue solid lines) and 200 nm (red dashed lines) represent the small fibers, while the cylinders with a radius of 1 μm (yellow dashed lines) and 1.5 μm (purple dashed lines) represent the coarse fibers in the tissue samples. Obviously, the phase functions confirm that the distribution of light scattered by the coarse cylinders (yellow and purple lines) is mainly concentrated in the range of small angles (15° corresponds to the scattering angle of 10×), or in other words, more photons are forward scattered by large fibers. For the objective lens, a large magnification means a large NA and an increased angle of reception for the scattered light. It can be clearly seen from Figure 3H that the diattenuation information induced by the coarse fibers is mainly concentrated in the forward scattering photons. That is to say, as the objective magnification or NA increases, the received scattering angle becomes larger, and more information about the small cylinders produce significant changes. As the objective magnification or NA increases, the received scattering angle becomes larger and the information from the tiny fibers is gradually blurred in the *D* image.

For a better explanation, the CSM-based Monte Carlo simulation was used to further analyze the relationship between scattering angle and parameter *D* value of cylinders with different sizes. Here, the simulation parameters are as follows: the radius of the cylinder scatterer is 100 nm, 200 nm, 1 μm, and 1.5 μm; the scattering coefficient is 200 cm^−1^; the refractive index is 1.43; the zenith angle is 90°; the cylinders are aligned in the x-y plane, and their orientations fluctuate around both the *x-* and *z-axis* following a Gaussian distribution with a standard deviation of 30° half-width. The refractive index of the medium is 1.35, the wavelength of the incident light is 633 nm, the thickness of the medium is 6 μm, and the number of incident photons is 10^7^.

It can be found in the MC simulation results in Figure 3J that 1) for the same scattering coefficient the *D* values of the small cylinders (blue and red lines) are larger than that of the coarse fibers (yellow and purple lines); 2) as the scattering angle increases from 5° to 55° the *D* values of the small cylinders decrease prominently, while the variations of the *D* values for large fibers are limited. The high NA objective also collects the photons’ information observed by the low NA one. However, the high NA objective receives light from a wider angle, which means that more photons are collected, and the information carried by small-angle photons is diluted by those received at larger angles. Therefore, the overall parameter *D* value becomes smaller. Both the MC simulation results and phase functions are shown in Figure 3 demonstrate that when a low magnification objective is used, the resolution is insufficient and only the coarse fibrous structures in the tissue can be observed. Since the diattenuation resulting from the coarse cylinder is smaller compared to that by the small fibers, a small mean parameter *D* value was observed at 4×. When the objective was changed from 4× to 10×, the information of small fibrous structures could be gradually detected, therefore the mean value of *D* increased. In the transition from 10× to 60×, the diattenuation brought by the fine fibers decreases as the magnification increases, and therefore the mean value of *D* decreases. The scatter-plot matrix in Figure 3I shows the general correlation among the Mean value of *D* under different magnification objectives (4×/NA 0.10, 10×/NA 0.25, 20×/NA 0.40, 40×/NA 0.65, and 60×/NA 0.80). The variables are written in a diagonal line from top left to bottom right. On the right of the diagonal is the correlation coefficient *R* between two pairs of the mean of *D* at different magnifications, with the larger the font size, the higher the correlation. On the left side of the diagonal is the scatter-plot, with smoother green trend lines indicating linear fitting for the data points from two pairs of the mean of *D* at different magnification lines to illustrate the underlying relationship. We can see from Figure 3I that the correlation between the mean values of the parameters *D* at different magnifications is not significant, with most of the *R* values ranging from 0.2 to 0.4, implying a moderate correlation. It indicates that the diattenuation information represented by parameter *D* is different at varying magnifications and different reception angles. It suggests that when using features of *D* images to distinguish between different fibrous structures, the relatively low-resolution images may serve as a better choice, such as 10×, which provides more information on fiber structure compared to high magnification together with fast imaging speed and wide FOV. As shown in Figure 2, the fibrous structures are clearer in *D* images compared to those in the *δ* and *Δ* images at low magnification. In contrast, the images of *δ* and *Δ* show clearer fiber structures at high magnification. It indicates that the influence of NA on polarization parameters is different. We can combine *D* images with other polarization parameter images to obtain comprehensive structural information about the sample. Recently, we developed an image fusion method based on color spaces to combine different polarimetric parameters to provide multi-dimensional structural information (Zhai et al., 2022). This method can improve the microstructural characterization at low magnification based on the parameters. Therefore, it may also be helpful to obtain clear *D* images by combining other polarization parameter images at high magnification for biomedical applications.

### Analysis of Linear Retardance

**FIGURE 4:**
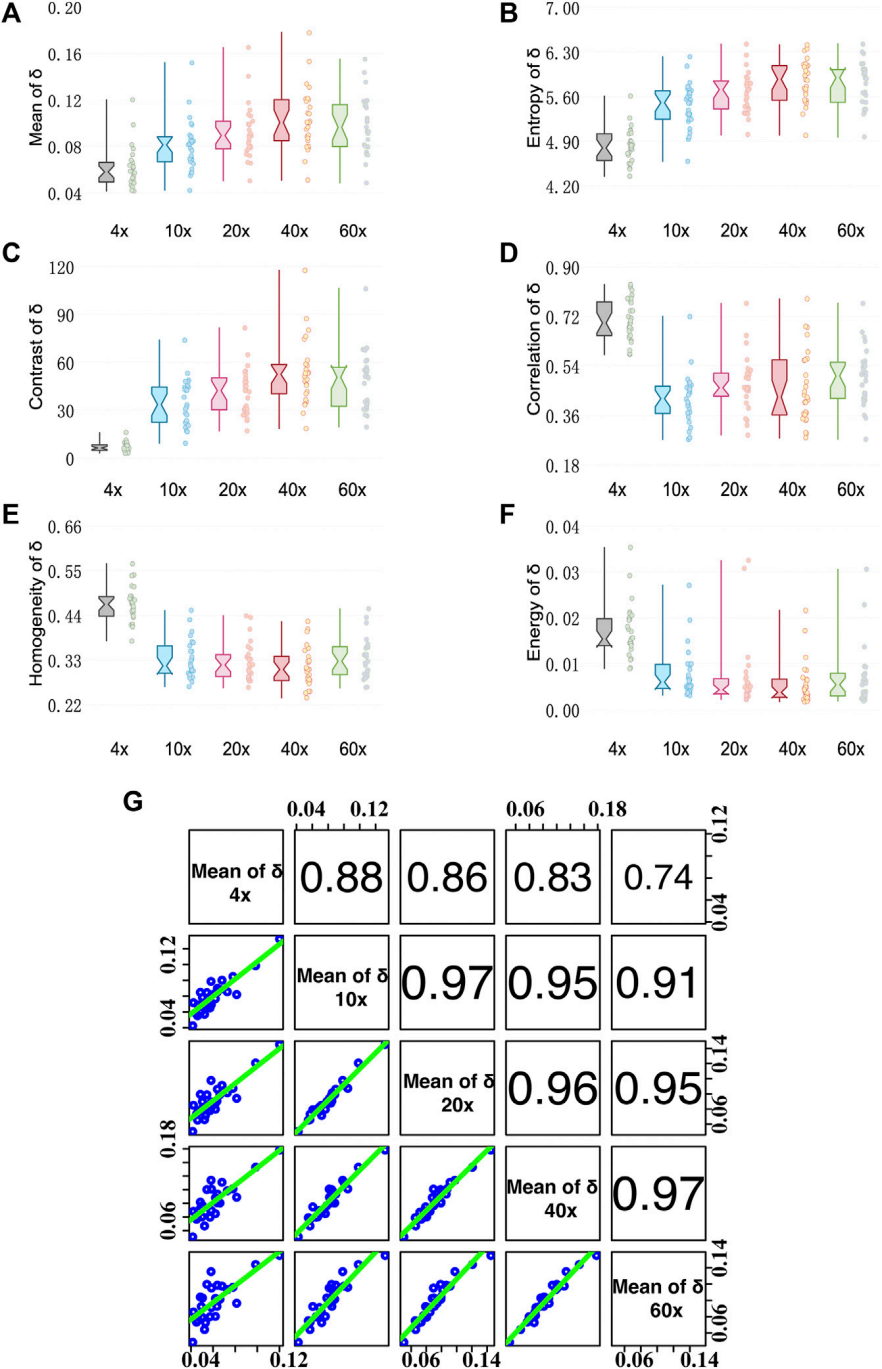
Quantitative analysis results of MMPD parameter *δ* images under different magnification objectives (4×/NA 0.10, 10×/NA 0.25, 20×/NA 0.40, 40×/NA 0.65, and 60×/NA 0.80). **(A–F)** Box plots of Mean, Entropy, Contrast, Correlation, Homogeneity, and Energy. **(G)** The Correlation coefficient *R* between two pairs of mean of *δ* at different magnifications. (cf. Figure 3I).

Figure 4 shows analysis results of MMPD parameter *δ* images. From Figure 4A we can see that the Mean value of the linear retardance *δ* gradually increases when the objective magnification increases from 4× to 40×, and then becomes stable when the magnification reaches 60×. The possible reason for such a changing trend is that the greater NA of the objective with a larger magnification means a larger receiving angle of the scattering photons, which have a longer propagation path in tissue compared with the ones of limited scattering numbers. Therefore, the more scattering photons contribute a larger value of linear retardance *δ*. We can also notice that the changing trend of Entropy shown in Figure 4B with the increasing objective magnification is similar to that of the Mean value.

For GLCM parameters, the Contrast value shown in Figure 4C gradually increases with the increase of the objective magnification, and the change becomes stable at 40× and 60×. It indicates that the *δ* image will be clearer when the objective of higher NA is used. Meanwhile, the other three GLCM parameters: Correlation shown in Figure 4D, Homogeneity shown in Figure 4E, and Energy shown in Figure 4F decrease as the magnification increases from 4× to 10×, then become relatively stable as the magnification changes. It indicates that the *δ* image texture is more uniform, similar, and ordered at a low magnification of 4×. The reason is that the loss of image details can lead to the image becoming smooth. However, when objective lenses of magnification larger than 10× are applied to the tissue sample, the texture feature information contained in *δ* images is stable. Moreover, as we can see in Figure 4G, Mean values of *δ* images have Pearson correlation coefficients R values > 0.7 for each objective at different magnifications, with a strong correlation with R values > 0.9 for objectives at 10× and higher magnifications. It demonstrates that the information of *δ* at high magnification can also be well observed at low magnification. The conclusion is in accordance with our previous study (Shen et al., 2020), which is the fibers density information contained in the texture features of the linear retardance *δ* parameter image is preserved well with the decline of imaging resolution.

### Analysis of Depolarization

**FIGURE 5:**
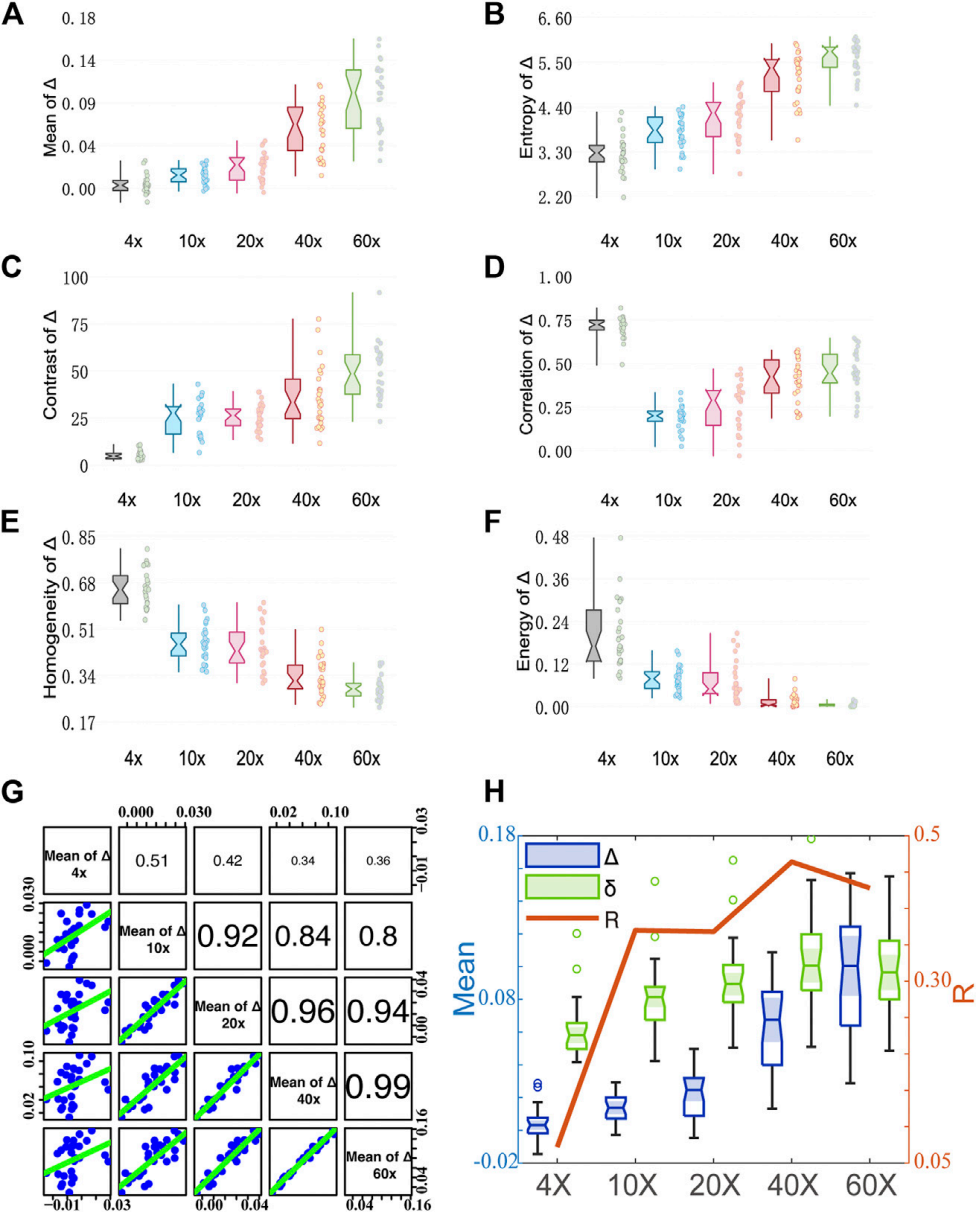
Quantitative analysis results of MMPD parameter *Δ* images under different magnification objectives (4×/NA 0.10, 10×/NA 0.25, 20×/NA 0.40, 40×/NA 0.65, and 60×/NA 0.80). **(A–F)** Box plots of Mean, Entropy, Contrast, Correlation, Homogeneity, and Energy. **(G)** The Correlation coefficient *R* between two pairs of the mean of *Δ* at different magnification objectives. (cf. Figure 3I). **(H)** The Correlation coefficient *R* between the mean of *Δ* and *δ*.

Figure 5 shows analysis results of MMPD depolarization parameter *Δ* images. As can be seen in Figures 5A,B, the larger magnification of the objective lens, the larger the Mean and Entropy values of *Δ* obtained. As the magnification of the objective becomes larger, more multiply scattered photons are collected, which contribute more to depolarization (Bicout et al., 1994; Brosseau, 1994; Aiello and Woerdman, 2005), leading to an increase in the observed Mean value of the MMPD *Δ* parameter. Also, the scattered photons contribute to the Entropy value of the *Δ* image. For GLCM parameters, first we can observe in Figure 5C that the larger magnification of the objective lens, the larger the Contrast value of *Δ* it shows. The increasing Contrast of the images means that the clarity of the *Δ* image increases significantly at high objective magnification. The values of Homogeneity shown in Figure 5E and Energy shown in Figure 5F are higher at small magnification. It means that the *Δ* image texture is more similar and ordered when the 4× objective is used. The Correlation shown in Figure 5D has a different trend from Homogeneity and Energy, where the value of 4× (0.5–0.8) is significantly higher than the other magnifications (0–0.6) It reflects the correlation of gray scale between pixels and spaced pixels. When the difference in gray values between pixels is small, the calculated correlation value is large. It means that the image texture uniformity is higher at 4×, the less fibrous structure can be observed and the texture looks more blurred, while more information on the fibrous structure can be obtained overall at higher magnification. Combining with Figure 2, we can find that under 4×, the image texture information is less, and the fiber structure is not obvious. As the resolution increases from 10× to 60×, the difference in gray value between the pixels of the fiber structure gradually decreases, therefore the Correlation of *Δ* gradually increases. However, the difference between fiber structure and non-fiber structure increases, resulting in the gradual decrease of Contrast-enhance, Homogeneity, and Energy. From Figure 5G, we can notice that the correlation of *Δ* images between 10× and higher magnification objectives is larger than 0.8. However, the correlation between 4× and other magnification objectives is relatively small. This also indicates that the information on fibrous structure at higher magnifications is better preserved in *Δ* images at 10×. If we want to obtain the information on fibrous structure from *Δ* images, a lower magnification, such as 10×, can be considered. It can be seen in Figures 4, 5, the trends of the first-order statistical moments, GLCM parameters of *δ* and *Δ* are similar. Some related literature (Wang and Wang, 2002; Alali, 2012; Ortega-Quijano et al., 2013; Pierangelo et al., 2013; He et al., 2015), indicated that *δ* can also induce depolarization in the scattering medium. Therefore, we correlate the variation processes of parameters *δ* and *Δ* with the magnification. Here, the correlation between Mean values of *δ* and *Δ* at different magnifications is plotted in Figure 5H. As the objective magnification increases, the receiving scattering angle increases, more multiply scattered photons that have longer propagation paths in tissue are collected, and the correlation between *δ* and *Δ* becomes larger, leading to larger *Δ* values induced by linear retardance.

## Conclusion

In this study, to analyze the influence of imaging resolution or objective magnification on polarization properties obtained from Mueller matrix microscopy, namely diattenuation, linear retardance, and depolarization, we measured the Mueller matrices of unstained rat dorsal skin tissue slices abundant in collagen fibers. We calculated MMPD parameters *D*, *δ,* and *Δ* images of the sections at a series of different objective magnifications with 4×/NA 0.10, 10×/NA 0.25, 20×/NA 0.40, 40×/NA 0.65, and 60×/NA 0.80. Then, we analyzed the first-order moments and GLCM image texture parameters in conjunction with MMPD parameters *D*, *δ,* and *Δ* images. The results show that 1) when using features of *D* images to distinguish between different fibrous structures, the relatively low-resolution images may serve as a better choice, such as 10×, which provide more information on fiber structure compared to high magnification together with fast imaging speed and wide FOV. 2) The information of *δ* at high magnification can also be well observed at low magnification, the fibers density information contained in the texture features of linear retardance *δ* parameter image is preserved well with the decline of imaging resolution. 3) The information of fibrous structures at higher magnifications is well preserved in *Δ* images at 10×. If we want to obtain the information on fibrous structures from *Δ* images, a lower magnification, such as 10×, can be considered. This study provides a criterion to decide which structural information can be accurately and rapidly obtained using a transmission Mueller matrix microscope with low NA objectives to assist pathological diagnosis and other applications.

## Data Availability

The raw data supporting the conclusion of this article will be made available by the authors, without undue reservation.
